# One-year outcomes in cardiogenic shock triggered by supraventricular tachycardia: an analysis of the FRENSHOCK multicenter prospective registry

**DOI:** 10.3389/fcvm.2023.1167738

**Published:** 2023-09-05

**Authors:** Miloud Cherbi, Eric Bonnefoy, Nicolas Lamblin, Edouard Gerbaud, Laurent Bonello, François Roubille, Bruno Levy, Sebastien Champion, Pascal Lim, Francis Schneider, Meyer Elbaz, Hadi Khachab, Jeremy Bourenne, Marie-France Seronde, Guillaume Schurtz, Brahim Harbaoui, Gerald Vanzetto, Nicolas Combaret, Vincent Labbe, Benjamin Marchandot, Benoit Lattuca, Caroline Biendel-Picquet, Guillaume Leurent, Etienne Puymirat, Philippe Maury, Clément Delmas

**Affiliations:** ^1^ Intensive Cardiac Care Unit, Rangueil University Hospital, Toulouse, France; ^2^ Institute of Metabolic and Cardiovascular Diseases (I2MC), UMR-1048, National Institute of Health and Medical Research (INSERM), Toulouse, France; ^3^ Intensive Cardiac Care Unit, Lyon Brom University Hospital, Lyon, France; ^4^ Urgences et Soins Intensifs de Cardiologie, CHU Lille, University of Lille, Inserm U1167, Lille, France; ^5^ Intensive Cardiac Care Unit and Interventional Cardiology, Hôpital Cardiologique du Haut Lévêque, Pessac, France; ^6^ Bordeaux Cardio-Thoracic Research Centre, U1045, Bordeaux University, Hôpital Xavier Arnozan, Pessac, France; ^7^ Cardiology Department, Hopital Nord, AP-HM, Aix-Marseille Université, Marseille, France; ^8^ Intensive Care Unit, Department of Cardiology, Assistance Publique-Hôpitaux de Marseille, Hôpital Nord, Marseille, France; ^9^ Mediterranean Association for Research and Studies in Cardiology (MARS Cardio), Marseille, France; ^10^ Cardiology Department, PhyMedExp, Université de Montpellier, INSERM, CNRS, INI-CRT, CHU de Montpellier, Montpellier, France; ^11^ Réanimation Médicale Brabois, CHRU Nancy, Vandoeuvre-les Nancy, France; ^12^ Anesthesiology and Intensive Care Department, Clinique de Parly 2, Ramsay Générale de Santé, Le Chesnay, France; ^13^ Univ Paris Est Créteil, INSERM, IMRB, Créteil, France; ^14^ Cardiology Department, AP-HP, Hôpital Universitaire Henri-Mondor, Service de Cardiologie, Créteil, France; ^15^ Médecine Intensive-Réanimation, Hôpital de Hautepierre, Hôpitaux Universitaires de Strasbourg, Strasbourg, France; ^16^ Intensive Cardiac Care Unit, Cardiology Department, CH d'Aix-en-Provence, Aix-en-Provence, France; ^17^ Service de Réanimation des Urgences, AP-HM, Hôpital de La Timone, Marseille, France; ^18^ Service de Cardiologie CHU, Besançon, France; ^19^ Cardiology Department, Hôpital Croix-Rousse and Hôpital Lyon Sud, Hospices Civils de Lyon, Lyon, France; ^20^ University of Lyon, CREATIS, UMR5220, INSERM U1044, INSA-15, Lyon, France; ^21^ Department of Cardiology, Hôpital de Grenoble, La Tronche, France; ^22^ Department of Cardiology, CHU Clermont-Ferrand, CNRS, Université Clermont Auvergne, Clermont-Ferrand, France; ^23^ Medical Intensive Care Unit, Assistance Publique-Hôpitaux de Paris (AP-HP), Hôpital Tenon, Paris, France; ^24^ Université de Strasbourg, Pôle D'Activité Médico-Chirurgicale Cardio-Vasculaire, Nouvel Hôpital Civil, Centre Hospitalier Universitaire, Strasbourg, France; ^25^ Department of Cardiology, Nîmes University Hospital, Montpellier University, Nîmes, France; ^26^ Department of Cardiology, CHU Rennes, Inserm, LTSI—UMR 1099, Univ Rennes 1, Rennes, France; ^27^ Department of Cardiology, Assistance Publique-Hôpitaux de Paris (AP-HP), Hôpital Européen Georges Pompidou, Paris, France; ^28^ Université de Paris, Paris, France; ^29^ REICATRA, Institut Saint Jacques, CHU de Toulouse, Toulouse France

**Keywords:** cardiogenic shock, supraventricular tachycardia, epidemiology, prognosis, mortality

## Abstract

**Background:**

Cardiogenic shock (CS) is the most severe form of heart failure (HF), resulting in high early and long-term mortality. Characteristics of CS secondary to supraventricular tachycardia (SVT) are poorly reported. Based on a large registry of unselected CS, we aimed to compare 1-year outcomes between SVT-triggered and non-SVT-triggered CS.

**Methods:**

FRENSHOCK is a French prospective registry including 772 CS patients from 49 centers. For each patient, the investigator could report 1–3 CS triggers from a pre-established list (ischemic, mechanical complications, ventricular/supraventricular arrhythmia, bradycardia, iatrogenesis, infection, non-compliance, and others). In this study, 1-year outcomes [rehospitalizations, mortality, heart transplantation (HTx), ventricular assist devices (VAD)] were analyzed and adjusted for independent predictive factors.

**Results:**

Among 769 CS patients included, 100 were SVT-triggered (13%), of which 65 had SVT as an exclusive trigger (8.5%). SVT-triggered CS patients exhibited a higher proportion of male individuals with a more frequent history of cardiomyopathy or chronic kidney disease and more profound CS (biventricular failure and multiorgan failure). At 1 year, there was no difference in all-cause mortality (43% vs. 45.3%, adjusted HR 0.9 (95% CI 0.59–1.39), *p* = 0.64), need for HTx or VAD [10% vs. 10%, aOR 0.88 (0.41–1.88), *p* = 0.74], or rehospitalizations [49.4% vs. 44.4%, aOR 1.24 (0.78–1.98), *p* = 0.36]. Patients with SVT as an exclusive trigger presented more 1-year rehospitalizations [52.8% vs. 43.3%, aOR 3.74 (1.05–10.5), *p* = 0.01].

**Conclusion:**

SVT is a frequent trigger of CS alone or in association in more than 10% of miscellaneous CS cases. Although SVT-triggered CS patients were more comorbid with more pre-existing cardiomyopathies and HF incidences, they presented similar rates of mortality, HTx, and VAD at 1 year, arguing for a better overall prognosis.

**Clinical Trial Registration:**

https://clinicaltrials.gov, identifier: NCT02703038.

## Graphical Abstract

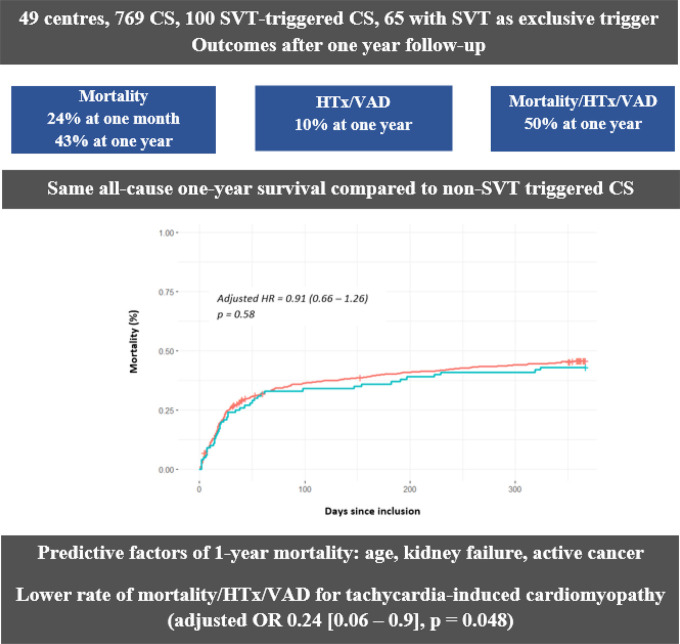
 The FRENSHOCK registry—1-year outcomes in cardiogenic shock triggered by supraventricular arrhythmia. CS, cardiogenic shock; HR, hazards ratio; HTx, heart transplantation; OR, odds ratio; SVT, supraventricular tachycardia; VAD, ventricular assist device

## Introduction

Cardiogenic shock (CS) is the most severe form of heart failure (HF), resulting in a life-threatening state of tissue hypoperfusion, which can lead to multiorgan failure and death (1). Despite recent improvements, the mortality rate remains extremely high, close to 50% in 1 year (2), depending on the underlying trigger (3).

The relationship between supraventricular tachycardia (SVT) and HF remains challenging. First, there is strong evidence suggesting that SVT is a poor prognostic factor in cases of chronic HF (4) or acute myocardial infarction (AMI) without CS (5).

Nevertheless, outside the context of CS (6, 7), (1) SVT is considered a negative prognostic marker in patients with altered ejection fraction and (2) the independent effect of SVT on mortality seems inversely related to the severity of HF, suggesting a potential role as a marker of advanced HF. In addition, the prognosis appears less affected for non-ischemic than ischemic heart disease in the case of new onset of SVT (8, 9). On the other hand, there are little available data regarding the short- and long-term outcomes of SVT-triggered CS, especially when it occurs without an acute ischemic trigger.

Hence, this study aimed to compare 1-year outcomes between SVT-triggered CS and non-SVT-triggered CS based on the multicenter prospective FRENSHOCK registry.

## Materials and methods

### Patient population

As previously reported (10), FRENSHOCK is a prospective, observational, and multicenter registry including 772 patients admitted between April and October 2016 for CS in the intensive care unit (ICU)/intensive cardiac care unit (ICCU) in France, coming from all types of institutions (primary to tertiary centers, university and non-university, and public and private hospitals).

All adult patients (≥18 years old) with CS were prospectively included in this registry if they met at least one criterion of each of the following three components: (1) low cardiac output: low systolic blood pressure (SBP) <90 mmHg, the need for maintenance with vasopressors/inotropes, or a low cardiac index <2.2 L/min/m²; (2) left and/or right heart filling pressure elevation, defined by clinical signs, radiology, blood tests, echocardiography, or signs of invasive hemodynamic overload; and (3) signs of organ malperfusion, which could be clinical (oliguria, confusion, pale and/or cold extremities, mottled skin) or biological (lactate > 2 mmol/L, metabolic acidosis, renal failure, liver insufficiency).

For each patient, the investigator could report 1–3 CS triggers from a pre-established list including ischemic (type 1 or 2 AMI), mechanical complications (valvular injury, ventricular septal defect), ventricular arrhythmia, supraventricular tachycardia, severe bradycardia, iatrogenesis (medication induced), infections, non-observance, or others. Hence, the SVT could be reported by the managing physician as the sole and exclusive trigger of CS (CS with SVT as an exclusive trigger) or be associated with one or two other coexisting triggers (CS with SVT as a non-exclusive trigger).

### Data collection

As previously described (3, 10), past medical history, ongoing treatments, and clinical, biological, and echocardiographic data were collected at admission and at 24 h. In-hospital CS management [especially inotropes/vasopressors, mechanical ventilation, renal replacement therapy, and short-term mechanical circulatory support (MCS)] was reported, as well as medication at admission, at discharge, and at 1 year. Precise mechanisms of SVT could not be retrieved from the database nor the evolution of SVT after adapted management.

### Outcomes

All-cause mortality, heart transplantation (HTx), and ventricular assist devices (VADs) were assessed at 1 month and 1 year. The primary endpoint was 1-year all-cause mortality. Secondary endpoints included 1-month all-cause mortality, 1-year need for HTx or VAD, the 1-year rate of cardiovascular rehospitalizations, and the composite of death, HTx, or VAD at 1 year. When done, SVT catheter ablation (11) and myocardial revascularization (12) were performed according to the current techniques.

### Ethics

The study was conducted in accordance with the Helsinki Declaration and French law. Written consent was obtained for all patients. Recorded data and their storage were approved by the CCTIRS (French Health Research Data Processing Advisory Committee) (no. 15.897) and the CNIL (French Data Protection Agency) (no. DR-2016-109).

### Statistical analysis

Continuous variables are reported as means ± SDs or medians and interquartile ranges (IQRs) when appropriate. Categorical variables are reported as frequencies and percentages. Comparisons were made using the Mann–Whitney non-parametric test for continuous variables and the chi-square test or Fisher's exact test for categorical variables. Paired data were analyzed with the Wilcoxon signed-rank test. Multivariate stepwise logistic regression analysis was performed to determine independent predictors for each primary and secondary outcome. First, the association of all baseline characteristics and each outcome of interest was assessed using univariable logistic regression analyses. Thereafter, all significant independent predictors were integrated into multivariable analyses for each outcome and backward reduced to only significant characteristics (*p* ≤ 0.05). Finally, these significant characteristics were incorporated in multivariable models as fixed covariates for each adjusted outcome analysis. The significant risk factors were reported with their respective odds ratios (ORs) and 95% confidence intervals (CIs). The variance inflation factor (VIF) was used to rule out multicollinearity among the variables. The primary outcome of all-cause mortality was assessed using Kaplan–Meier time-to-event analysis, and the adjusted hazard ratios (HRs), 95% CIs, and *p*-values were determined by Cox proportional hazards models. Secondary outcomes (HTx, VAD, and further composites) are reported as their adjusted ORs and 95% CIs.

The main analysis was a comparison between SVT-triggered and non-SVT-triggered CS. Further analyses were conducted about the primary and secondary endpoints in the SVT-triggered group between patients with SVT as an exclusive trigger and those with other coexisting triggers, as well as between CS with SVT as exclusive trigger without a history of cardiomyopathy (CM) and other SVT-triggered CS.

Analyses were performed using R software [version 4.1.2 (2021-11-01)]. All tests were two-tailed. A value of *p* ≤ 0.05 was considered statistically significant.

## Results

### Overall population

**Figure 1:**
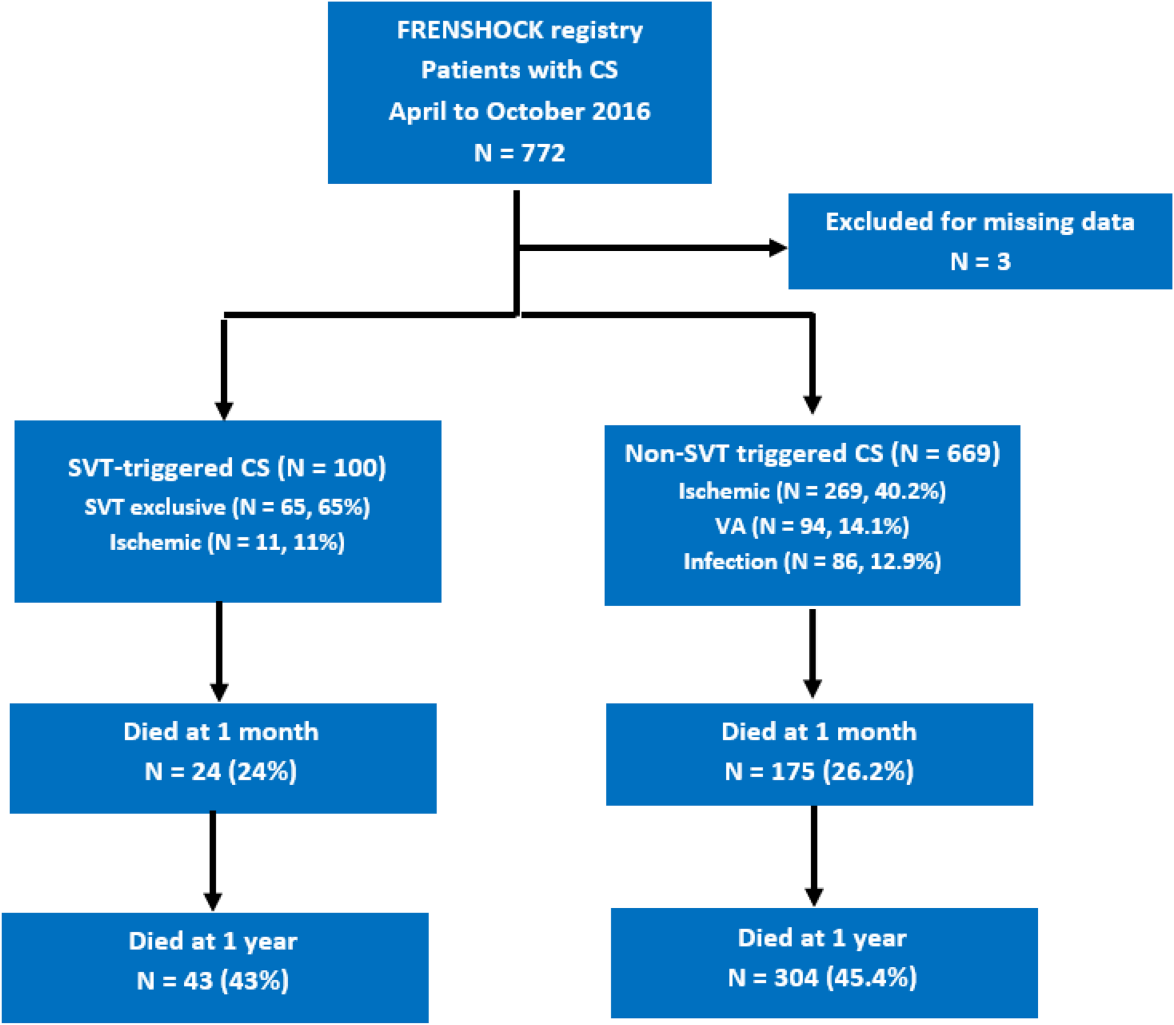
Flowchart of the study. CS, cardiogenic shock; SVT, supraventricular tachycardia; VA, ventricular arrythmias.

After the exclusion of three patients for missing data, 769 patients were included in 49 centers (Figure 1). Table 1 reports the initial characteristics of included patients. Patients were predominantly men (71.4%) with a mean age of 65.8 ± 14.8 years. Previously known heart disease was reported for 56% (29.9% ischemic, 10% dilated, and 8.5% valvular) patients, most of whom had a New York Heart Association (NYHA) stage II or III (26% or 26.4%, respectively), consistent with a substantial rate of chronic heart failure treatments (41.1%, 37.9%, and 13.8% for beta blockers, ACEi/ARB, and aldosterone antagonists, respectively). Table 2 summarizes the initial clinical, biological, and echocardiographic data. The mean MBP was 74.9 ± 18.4 mmHg, with initial cardiac arrest for 78 patients (10.2%). The mean left ventricle ejection fraction (LVEF) was 26.3 ± 13.4%, with a median tricuspid annular plane systolic excursion (TAPSE) of 13 mm (10–16) and a median peak systolic velocity tissue Doppler imaging (PSVtdi) of 8 cm/s (6–11).

**Table 1:** Baseline characteristics at admission according to cardiogenic shock triggers (SVT vs. non-SVT).

	Overall population	SVT-triggered CS	Non-SVT-triggered CS	*p*-value
	(*n* = 769)	(*n* = 100)	(*n* = 669)	
Age, mean ± SD, years	65.8 ± 14.8	66 ± 12.5	65.7 ± 15.1	0.59
Male, *n* (%)	549 (71.4)	81 (81)	468 (70)	0.03
Body mass index, mean ± SD, kg/m²	25.9 ± 5.5 (*n* = 741)	26.5 ± 5.2 (*n* = 99)	25.8 ± 5.6 (*n* = 642)	0.16
Risk factors, *n* (%)				
Diabetes mellitus	217 (28.3) (*n* = 767)	27 (27)	190 (28.5) (*n* = 667)	0.85
Hypertension	363 (47.3) (*n* = 768)	46 (46)	317 (47.5) (*n* = 668)	0.87
Dyslipidemia	277 (36.1) (*n* = 768)	37 (37)	240 (35.9) (*n* = 668)	0.92
Current smoker	205 (27.8) (*n* = 77)	26 (26)	179 (28.1) (*n* = 637)	0.75
Medical history, *n* (%)				
Chronic kidney failure	163 (21.2) (*n* = 768)	32 (32)	131 (19.6) (*n* = 668)	<0.01
ICD	127 (16.5) (*n* = 768)	23 (23)	104 (15.6) (*n* = 668)	0.08
Active cancer	51 (6.6) (*n* = 768)	5 (5)	46 (6.9) (*n* = 668)	0.62
Stroke	62 (8.1) (*n* = 768)	14 (14)	48 (7.2) (*n* = 668)	0.03
History of cardiac disease, *n* (%)				
All causes	430 (56.0) (*n* = 768)	77 (77)	353 (52.8) (*n* = 668)	<0.01
Ischemic	230 (29.9) (*n* = 768)	39 (39)	191 (28.6) (*n* = 668)	0.045
Toxic	33 (4.3) (*n* = 768)	7 (7)	26 (3.9) (*n* = 668)	0.24
Dilated	77 (10) (*n* = 768)	15 (15)	62 (9.3) (*n* = 668)	0.11
Valvular	65 (8.5) (*n* = 768)	15 (15)	50 (7.5) (*n* = 668)	0.02
NYHA functional status, *n* (%)				
I	263 (3.5) (*n* = 750)	16 (16.2) (*n* = 99)	247 (37.9) (*n* = 651)	<0.01
II	195 (26.0) (*n* = 750)	36 (36.4) (*n* = 99)	159 (2.4) (*n* = 651)	
III	198 (26.4) (*n* = 750)	35 (35.4) (*n* = 99)	163 (25) (*n* = 651)	
IV	94 (12.5) (*n* = 750)	12 (12.1) (*n* = 99)	82 (12.6) (*n* = 651)	
Previous medications, *n* (%)				
P2Y12 inhibitors	126 (16.4) (*n* = 767)	10 (10)	116 (17.4) (*n* = 667)	0.09
Vitamin K antagonist	163 (21.2) (*n* = 767)	32 (32)	131 (19.6) (*n* = 667)	<0.01
DOAC	56 (7.3) (*n* = 767)	22 (22)	34 (5.1) (*n* = 667)	<0.01
ACE inhibitors	291 (37.9) (*n* = 767)	43 (43)	248 (37.2) (*n* = 667)	0.31
Sacubitril/valsartan	17 (2.3) (*n* = 724)	4 (4.2) (*n* = 95)	13 (2.1) (*n* = 629)	0.36
Betablockers	315 (41.1) (*n* = 767)	51 (51)	264 (40) (*n* = 667)	0.04
Loop diuretics	373 (48.6) (*n* = 767)	65 (65)	308 (46.2) (*n* = 667)	<0.01
Aldosterone antagonists	106 (13.8) (*n* = 767)	26 (26)	80 (12) (*n* = 667)	<0.01
Amiodarone	130 (17.4) (*n* = 749)	35 (35.4) (*n* = 99)	95 (14.6) (*n* = 650)	<0.01

ACE, angiotensin-converting enzyme; ICD, implantable cardioverter-defibrillator; NYHA, New York Heart Association; SD, standard deviation.

**Table 2:** Clinical, echocardiographic, and laboratory parameters according to cardiogenic shock triggers (SVT vs non-SVT).

	Overall population	SVT-triggered CS	Non-SVT-triggered CS	*p*-value
	(*n* = 769)	(*n* = 100)	(*n* = 669)	
Clinical presentation at admission				
SBP, mean ± SD, mmHg	101.3 ± 25.2 (*n* = 767)	103.7 ± 27.7	100.9 ± 24.8 (*n* = 667)	0.38
DBP, mean ± SD, mmHg	63.2 ± 17.4 (*n* = 766)	65.8 ± 18.7 (*n* = 99)	62.9 ± 17.2 (*n* = 667)	0.14
MBP, mean ± SD, mmHg	74.9 ± 18.4 (*n* = 764)	78.3 ± 20.9 (*n* = 99)	74.4 ± 17.9 (*n* = 665)	0.13
Sinus rhythm, *n* (%)	398 (52.0) (*n* = 765)	14 (14)	384 (57.7) (*n* = 665)	<0.01
Cardiac arrest, *n* (%)	78 (10.2) (*n* = 768)	7 (7)	71 (10.6) (*n* = 668)	0.35
Blood tests at admission, median (IQR)				
Sodium, mmol/L	135 (132–139) (*n* = 757)	135.5 (132–139)	135 (132–139) (*n* = 657)	0.97
Potassium, mmol/L	4 (4–5) (*n* = 635)	4.38 (4–5) (*n* = 82)	4 (4–5) (*n* = 553)	0.13
Creatinin, μmol/L	133 (96–189.5) (*n* = 758)	143 (109.8–210.3)	131 (93.3–181.8) (*n* = 658)	<0.01
Bilirubin, mg/L	16 (9–29) (*n* = 541)	24.5 (16.3–40.8) (*n* = 78)	15 (9–26) (*n* = 463)	<0.01
Hemoglobin, g/dl	12.6 (11–14) (*n* = 751)	13 (11.5–14.8) (*n* = 99)	12.3 (11–14) (*n* = 652)	0.02
PT, %	59 (37–77) (*n* = 728)	46 (29.5–63.5) (*n* = 99)	61 (39–78) (*n* = 629)	<0.01
Nt-proBNP, pg/ml	9,516 (4,064–22,149) (*n* = 221)	12,300 (6,554–20,737) (*n* = 29)	8,380 (3,644–22,702.5) (*n* = 192)	0.11
BNP, pg/ml	1,150 (476.8–2,757.3) (*n* = 264)	1,417 (651.5–2,689) (*n* = 31)	1,142 (467–2,747) (*n* = 233)	0.5
Baseline echocardiography				
LVEF, mean ± SD, %	26.3 ± 13.4 (*n* = 760)	23.8 ± 12.1 (*n* = 98)	26.7 ± 13.5 (*n* = 662)	0.04
TAPSE, median (IQR), mm	13 (10–16) (*n* = 257)	11 (10–13) (*n* = 37)	14 (10–17) (*n* = 220)	0.01
PSVtdi, median (IQR), cm/s	8 (6–11) (*n* = 205)	7 (6–8.5) (*n* = 35)	8.5 (6–11) (*n* = 170)	0.04
Severe mitral regurgitation, *n* (%)	106 (14.52) (*n* = 730)	22 (22.7) (*n* = 97)	84 (13.3) (*n* = 633)	0.02

BNP, brain natriuretic peptide; DBP, diastolic blood pressure; IQR, interquartile range; MBP, mean blood pressure; Nt-proBNP, N-terminal-pro hormone BNP; PT, prothrombin time.

Among the 769 CS patients, 100 were SVT-triggered (13%), of which 65 (8.5%) were exclusively triggered by SVT. Associated triggers reported in the SVT-CS group were ischemic (11%), iatrogenesis (7%), and infectious disease (6%) (Table 3). By contrast, among the 669 non-SVT-triggered CS patients, the main triggers were ischemic (40.2%), ventricular arrhythmia (14.1%), and infectious disease (12.9%).

**Table 3:** Distribution of cardiogenic shock triggers between groups.

	SVT-triggered CS	Non-SVT-triggered CS
	(*n* = 100)	(*n* = 669)
Ischemic, *n* (%)	11 (11)	269 (40.2)
Mechanical complications, *n* (%)	1 (1)	23 (3.4)
Ventricular arrhythmia, *n* (%)	0 (0)	94 (14.1)
Conduction disorder, *n* (%)	1 (1)	17 (2.5)
Infectious disease, *n* (%)	6 (6)	86 (12.9)
Non-observance, *n* (%)	5 (5)	22 (3.3)
Iatrogenesis, *n* (%)	7 (7)	40 (6)

### CS presentation and evolution at 24 h according to SVT and non-SVT groups

As reported in Table 1, initially, SVT-triggered CS patients exhibited higher proportions of male individuals (81% vs. 70%, *p* = 0.03), chronic kidney disease (32% vs. 19.6%, *p* < 0.01), and a history of previous heart disease (77% vs. 52.8%, *p* < 0.01), with an emphasis on ischemia (39% vs. 28.6%, *p* = 0.045) and valvular heart disease (15% vs. 7.5%, *p* = 0.02). Treatments with loop diuretics (65% vs. 46.2%, *p* < 0.01), aldosterone antagonists (26% vs. 12%, *p* < 0.01), amiodarone (35.4% vs. 14.6%, *p* < 0.01), vitamin K antagonist (VKA) (32% vs. 19.6%, *p* < 0.01), and direct oral anticoagulant (DOAC) (22% vs. 5.1%, *p* < 0.01) were significantly more commonly used in the SVT group.

SVT-triggered CS patients presented initially with higher initial creatinine and bilirubin levels, lower prothrombin time, lower LVEF, TAPSE, and PSVtdi, and more frequent severe mitral regurgitation (Table 2). After 24 h of management, the recovery was significantly better and more complete in the non-SVT group, as illustrated by a significant improvement in blood pressure, creatinine, bilirubin, lactate, and left ventricular ejection fraction parameters (Supplementary Table S1). At the time of initial care, 14% of patients in the SVT-triggered group presented with sinus rhythm against 57.7% of patients in the non-SVT group (*p* < 0.01).

### In-hospital management according to SVT and non-SVT groups

As summarized in Table 4, inotropes were used in 89.8% of the overall population, with more frequent use of norepinephrine in the non-SVT group (42% vs. 55.2%, *p* = 0.02) and levosimendan in the SVT-triggered group (13% vs. 6.6%, *p* = 0.04). No between-group difference was found for ventilation, renal replacement therapy, and mechanical circulatory support.

**Table 4:** In-hospital management according to cardiogenic shock triggers (SVT vs non-SVT).

	Overall population	SVT-triggered CS	Non-SVT-triggered CS	*p-*value
	(*n* = 769)	(*n* = 100)	(*n* = 669)	
Medications used, *n* (%)				
Dobutamine or norepinephrine or levosimendan	687 (89.8) (*n* = 765)	87 (87)	600 (90.2) (*n* = 665)	0.41
Dobutamine	629 (82.2) (*n* = 765)	79 (79)	550 (82.7) (*n* = 665)	0.45
Norepinephrine	409 (53.5) (*n* = 765)	42 (42)	367 (55.2) (*n* = 665)	0.02
Levosimendan	57 (7.5) (*n* = 765)	13 (13)	44 (6.6) (*n* = 665)	0.04
Respiratory support, *n* (%)				
Non-invasive	199 (26.0) (*n* = 765)	23 (23)	176 (26.5) (*n* = 665)	0.54
Invasive	290 (37.9) (*n* = 765)	31 (31)	259 (38.9) (*n* = 665)	0.16
Short-term mechanical circulatory support, *n* (%)				
IABP	48 (6.3) (*n* = 765)	3 (3)	45 (6.8) (*n* = 665)	0.22
Impella	26 (3.4) (*n* = 765)	3 (3)	23 (3.5) (*n* = 665)	1
ECLS	84 (11.0) (*n* = 766)	12 (12)	72 (10.8) (*n* = 666)	0.85
Renal replacement therapy, *n* (%)	122 (15.9) (*n* = 768)	14 (14)	108 (16.2) (*n* = 668)	0.68

ECLS, extracorporeal life support; IABP, intra-aortic balloon pump.

### Antiarrhythmic therapy

Table 5 describes the antiarrhythmic therapy used in our population. Beta blockers and amiodarone were more frequently used in the SVT-triggered group at initial care (51% vs. 40%, *p* = 0.04 and 35.4% vs. 14.6%, *p* < 0.01), although at 24 h at discharge, only amiodarone was more frequently used in the SVT-triggered group (54% vs. 29.5%, *p* < 0.01, and 47.1% vs. 22.7%, *p* < 0.01).

**Table 5:** Antiarrhythmic therapies according to cardiogenic shock triggers (SVT vs non-SVT).

	Overall population	SVT-triggered CS	Non-SVT-triggered CS	*p-*value
	(*n* = 769)	(*n* = 100)	(*n* = 669)	
Betablockers, *n* (%)				
Initial care	315 (41.1) (*n* = 767)	51 (51)	264 (40) (*n* = 667)	0.04
24 h	95 (13.8) (*n* = 690)	13 (14) (*n* = 93)	82 (13.7) (*n* = 597)	1
Discharge	306 (56.0) (*n* = 546)	41 (57.7) (*n* = 71)	265 (55.8) (*n* = 475)	0.86
1 year	235 (65.1) (*n* = 361)	28 (59.6) (*n* = 47)	207 (65.9) (*n* = 314)	0.49
Amiodarone, *n* (%)				
Initial care	130 (17.4) (*n* = 749)	35 (35.4) (*n* = 99)	95 (14.6) (*n* = 650)	<0.01
24 h	228 (46.0) (*n* = 496)	54 (54) (*n* = 92)	174 (29.5) (*n* = 590)	<0.01
Discharge	137 (25.8) (*n* = 531)	32 (47.1) (*n* = 68)	105 (22.7) (*n* = 463)	<0.01
1 year	57 (17.2) (*n* = 331)	12 (28.6) (*n* = 42)	45 (15.6) (*n* = 289)	0.06
ICD implantation, *n* (%)	37 (5.1) (*n* = 731)	4 (4.4) (*n* = 91)	33 (5.2) (*n* = 640)	0.96
SVT catheter ablation, *n* (%)	16 (2.2) (*n* = 731)	10 (11) (*n* = 91)	7 (1.1) (*n* = 640)	<0.01

SVT catheter ablation was performed in 10 patients of the SVT group vs. seven patients of the non-SVT group during initial CS hospitalization (11% vs. 1.1%, *p* < 0.01) because of the occurrence of SVT after inclusion in this group.

### Short- and long-term outcomes

**Figure 2:**
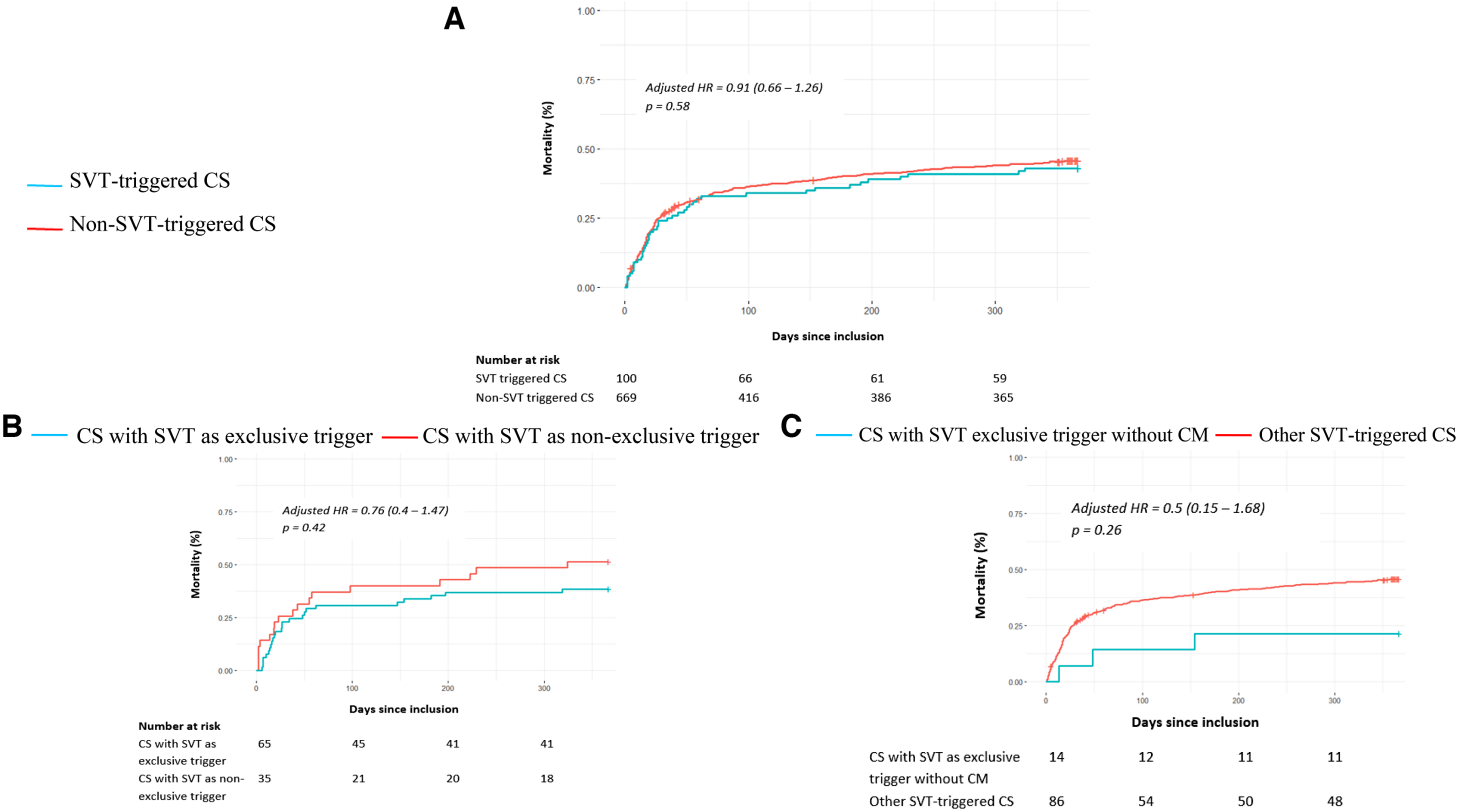
One-year all-cause mortality in patients with cardiogenic shock according to a supraventricular tachycardia trigger. The primary outcome of 1-year overall mortality is presented for SVT- and non-SVT-triggered CS patients (**A**), for CS patients with SVT as an exclusive trigger and CS patients with SVT and coexisting triggers (**B**), and for tachycardia-induced cardiomyopathy (CS with SVT as exclusive trigger and without previous cardiomyopathy) and other SVT-triggered CS patients (**C**). The cumulative incidences of 1-year and 1-month mortality were estimated using the Kaplan–Meier method; hazard ratios and 95% confidence intervals were estimated using Cox regression models. For all panels, 1-year all-cause mortality was adjusted for age, chronic kidney failure, and active cancer according to significant characteristics found as independent predictive factors in multivariable analyses.

**Figure 3:**
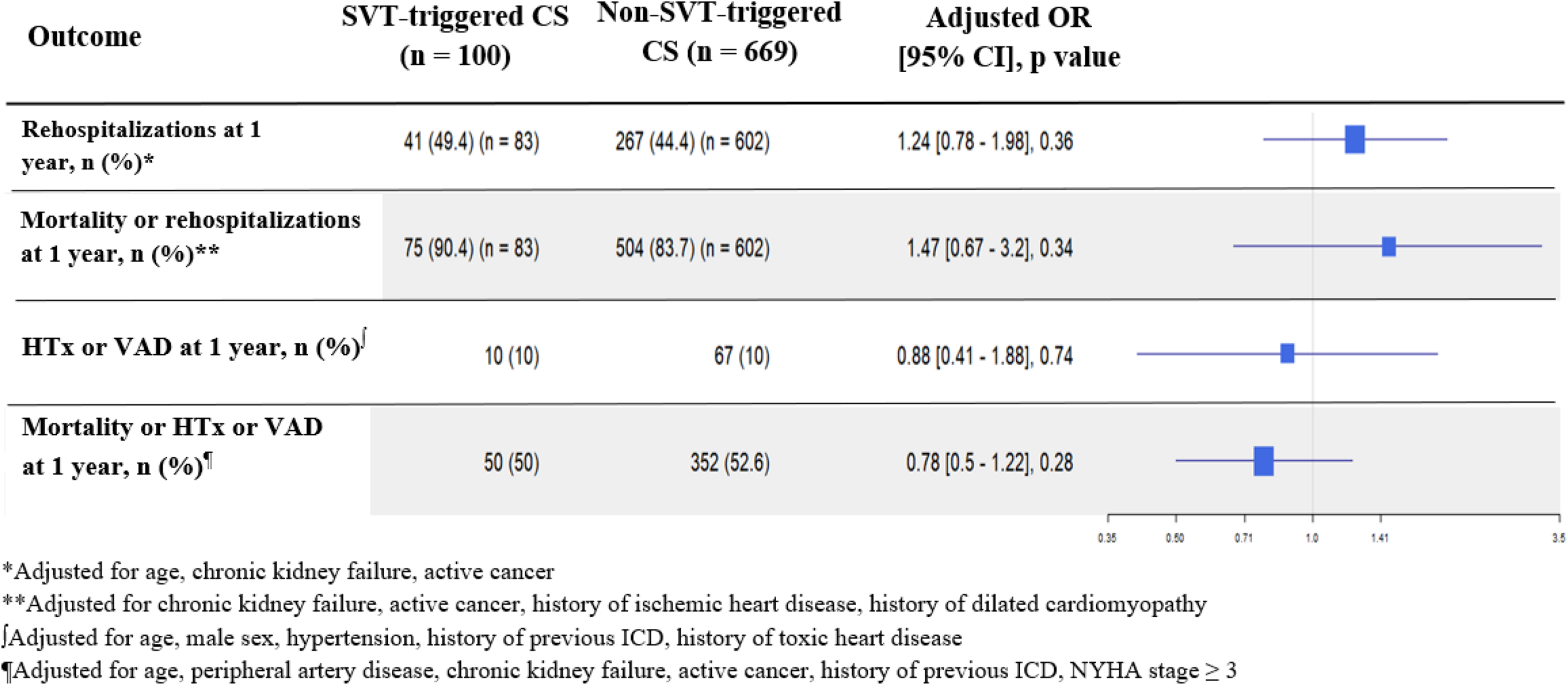
Secondary outcomes of rehospitalizations, heart transplantation, and ventricular assist devices in the overall population. Each adjusted outcome analysis included significant characteristics found as independent predictive factors in multivariable analyses and used as fixed covariates.

Figure 2 shows the absence of a 1-year all-cause mortality difference between SVT- and non-SVT-triggered CS [43% vs. 45.3%, adjusted HR of 0.9 (95% CI 0.59–1.39), *p* = 0.64]. The same results were found for 1-month all-cause mortality [24% vs. 26.2%, adjusted HR of 0.91 (95% CI 0.66–1.26), *p* = 0.58] (Figure 1). As reported in Figure 3, no difference was found in any secondary outcomes for cardiovascular rehospitalization, HTx, and VAD.

### SVT-triggered cardiogenic shocks

Among the 100 SVT-triggered CS patients, 65 presented initially with SVT as an exclusive trigger (distribution reported in Table 3), with balanced baseline characteristics between groups (Supplementary Table S2), except for higher rates of active cancers in the non-SVT-exclusive group (0% vs. 14.3%, *p* < 0.01), and aspirin treatment, more frequent in the SVT-exclusive group (46.2% vs. 20%, *p* = 0.02). The SVT-exclusive group presented with higher diastolic and mean blood pressure, with no difference in any biological or echocardiographic parameters except for higher sodium in the SVT-exclusive group (Supplementary Table S3). After 24 h, the exclusive SVT group showed a faster onset of LVEF recovery, while the non-SVT-exclusive group exhibited a more rapid decrease in lactate levels. In both groups, no substantial improvement was observed in blood pressure or renal and hepatic functions (Supplementary Table S4). Significant associations between baseline characteristics and each outcome of interest can be found in Supplementary Table S5.

**Figure 4:**
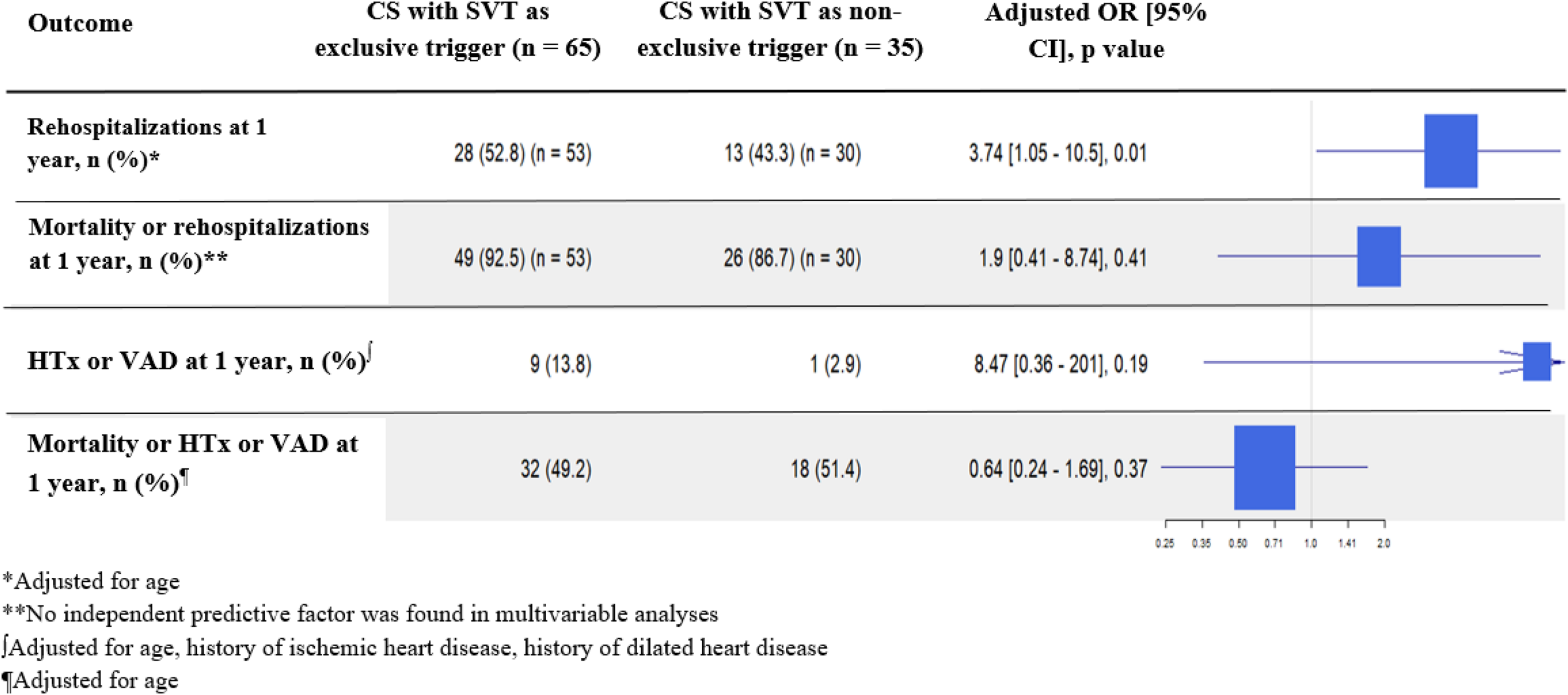
Secondary outcomes of rehospitalizations, heart transplantation, and ventricular assist devices in the SVT-triggered group. Each adjusted outcome analysis included significant characteristics found as independent predictive factors in multivariable analyses and used as fixed covariates.

Survival analyses did not show a difference in all-cause mortality at 1 month [adjusted HR 0.72 (95% CI 0.3–1.69), *p* = 0.45] and 1 year [adjusted HR 0.76 (95% CI 0.4–1.47), *p* = 0.42] (Figure 2) between SVT-exclusive and non-SVT-exclusive groups. As reported in Figure 4, no difference was observed for all secondary outcomes except for a higher rate of 1-year cardiovascular rehospitalizations in the SVT-exclusive group with an adjusted OR of 3.74 (95% CI 1.05–10.5, *p* = 0.01).

All data relating to in-hospital management are reported in Supplementary Table S6. No difference was found in using any antiarrhythmic drug, neither at admission nor at 24 h, at discharge, or at 1 year (Supplementary Table S7).

### CS with SVT as an exclusive trigger without a history of CM

Fourteen of the 100 SVT-triggered CS patients met this definition. As reported in Figure 2 and Supplementary Figure S1, overall composite criteria combining 1-year rates of mortality or HTx or VAD revealed a better outcome in this group with an adjusted OR of 0.23 (95% CI 0.04–0.95, *p* = 0.048).

## Discussion

To date, FRENSHOCK is the largest European prospective, observational, multicenter registry on CS, representing a real-world cohort from a broad spectrum of etiologies, including a relevant number of SVT-triggered CS patients, mostly non-ischemic, differing from previous surveys.

Analysis of the relationship between arrhythmia triggers and outcomes in unselected CS is scarce in the literature. We previously reported that ventricular arrhythmia is a common trigger of CS (12% in the FRENSHOCK population) associated with similar high mortality to other etiologies of CS but resulted in more heart transplantation and VAD cases at 1 year, especially in non-ischemic cardiomyopathy, suggesting the need for earlier evaluation by advanced heart failure specialized teams for a possible indication of mechanical circulatory support or heart transplantation (11).

Based on the FRENSHOCK registry, we would like to address the relationship between SVT and CS presentation, management, and outcomes.

Outside the setting of CS, several studies demonstrated that the presence of SVT (symptomatic or not) in patients with HF is associated with an increased risk for all-cause mortality, explained mainly by an increased risk for pump-failure death, suggesting that SVT may have a role in accelerating myocardial decline (7). By contrast, other studies, including, on average, patients with more severe HF, agreed not to support the concept that the presence of SVT in patients with advanced chronic HF is independently related to an adverse outcome during a long-term follow-up, considering SVT as a marker of advanced HF (6).

In this study, despite an initial presentation marked by more acute kidney and hepatic injuries and more severe biventricular dysfunction, SVT-triggered CS presented a similar 1-month rate of all-cause mortality to non-SVT-triggered CS. In addition, after 1 year of follow-up, no difference was observed in mortality, HTx or VAD, and rehospitalizations, suggesting a faster recovery in the medium and long term once the acute phase is resolved, indicative of an overall better prognosis.

Notwithstanding the high prevalence of SVT in CS, little has been reported about how they influence short- and long-term prognosis. Primary available data dealing with SVT and CS refer to the occurrence of arrhythmia in the case of CS complicating AMI, representing a minority in our cohort (11% of the 100 SVT-triggered CS patients), with no increase in 1-month and 1-year mortality (12, 13). A recent single-center retrospective study, including 222 patients with CS [of which 40 presented atrial fibrillation (AF)], focused on new-onset AF, indicating that although the presence of this arrhythmia can have a hemodynamic impact, it does not influence mortality rates (14), consistent with our results. As the relationship between SVT and ischemic heart disease is now well documented, further studies could focus on the influence of SVT-triggered CS in specific non-ischemic cardiomyopathies (e.g., dilated, hypertrophic, restrictive).

In comparison to the set of all CS triggers, pejorative independent predictive factors for 1-year all-cause mortality in the SVT-triggered CS population were age, chronic kidney failure, and active cancer, with variable correlation with other CS surveys such as the FAST-MI registry, which also highlighted age and history of kidney disease (15) or the CardShock study (16), underlying AMI, age, previous myocardial infarction, or prior coronary artery bypass as short-term mortality predictors. Yet, several studies found a higher mortality rate in non-ischemic heart disease (17). In our SVT-triggered CS group, coexisting ischemic trigger was not an independent pejorative predictive factor for mortality.

When exclusively triggered by SVT, the post-CS 1-year follow-up revealed a higher rate of cardiovascular rehospitalizations, consistent with many previous publications showing strong evidence for a high 30-day rate of rehospitalizations in the case of SVT and HF (18, 19). Even if no difference was found in mortality, HTx, or VAD, this trend should be highlighted, given the economic burden of rehospitalizations for SVT, which is probably underestimated (20). This trend leads us to assume that SVT can sometimes be considered a marker of myocardial decline, indicating a progression through the cascade of disease severity. Nonetheless, in our study, rehospitalizations were recorded globally from all cardiovascular causes without information on the possible recurrence of SVT.

To avoid the risk of misclassification, further analyses were made focusing on CS when exclusively triggered by SVT, without any additional trigger, as well as when occurring without a history of heart disease. Fourteen patients had an exclusively SVT-triggered CS occurring without previous heart disease and were associated with a significantly lower rate of the overall composite criteria combining 1-year mortality, HTx, and VAD. Even though we did not have enough data to sort them clearly, it might be in this part of the population that patients with tachycardia-induced cardiomyopathy (TIC), a clinical condition in which a persistent tachyarrhythmia or frequent ectopy contributes to ventricular dysfunction leading to systolic heart failure (21), are found. In addition, even if it should be taken with caution given the low number of patients, better outcome of 1-year mortality, HTx, or VAD seems consistent with previous studies, emphasizing restoration of LV function and reversal of LV remodeling with successful elimination of tachycardia in the majority of patients (22), even in emergency cases (23).

The relationship between SVT and advanced HF remains challenging, sometimes leading to iterative recurrences of CS because of inefficient maintenance of sinus rhythm, possibly requiring circulatory support and/or heart transplantation (24). Further studies could focus on patients with extremely severe SVT-triggered CS fulfilling the criteria for urgent indication of HTx and the prospect of escaping it through the restoration of sinus rhythm by efficient ablation.

### Limitations

First, from available data, we were not able to distinguish between different subtypes of atrial arrhythmia (e.g., atrial fibrillation, flutter, focal tachycardia) and their classification (first diagnosed, permanent, persistent, paroxysmal), although they fall under different management practices and could lead to different outcomes (25). Another main limitation was the assessment of return to sinus rhythm, which was only available during initial care and at discharge, limiting specific considerations, while long-term maintenance of sinus rhythm appears associated with better outcomes (26, 27). However, within the SVT-triggered group, we found the same rates of pharmacological and invasive antiarrhythmic treatments, suggesting that whatever the arrhythmia subtype and its curative strategy were, we achieved a good balance between groups. Furthermore, we had no information about SVT duration before CS, which might be a determining criterion for management strategy. Indeed, there is a singular difference between recent new-onset SVT, for which treatment should be to terminate SVT and prevent future recurrences using antiarrhythmic drugs and/or electrical cardioversion, followed by catheter ablation if needed, and chronic permanent SVT with high ventricular rate, less likely to be successfully converted and maintained in sinus rhythm, with a similar profile to that of end-stage heart failure. Future work on this topic should highlight this nuance, which was not detailed enough in this study.

While the crucial role of catheter ablation of SVT in heart failure is currently accepted (26, 28), only 11% of patients from the SVT-triggered CS benefited from such a procedure in our survey. Indeed, on top of including general hospitals with fewer facilities for carrying out an ablation, the cohort was conducted in 2016, when this type of procedure was less commonly performed than today. Ideally, this analysis should be done again with current data, and probably better outcomes would be observed in SVT-triggered CS.

Although considering all-cause mortality as the primary outcome was an intentional choice, since it represents the daily reality of the numerous comorbidities of patients suffering heart failure, future studies could also focus on specific cardiovascular outcomes and figure out a difference with all-cause mortality.

As previously reported (3), the FRENSHOCK registry involves risks of selection bias related to non-consecutive inclusions or exclusion of the most severe cases, with specific inclusion and exclusion criteria limiting the applicability to all patients with CS. We were not able to use the SCAI SHOCK Stage Classification, given that it was not yet available at the time of our study.

## Conclusion

SVT is a frequent trigger of CS alone or in association. Although SVT-triggered CS patients were more comorbid with more pre-existing cardiomyopathies and HF incidences, they presented similar rates of mortality, HTx, and VAD at 1 year, arguing for a better overall prognosis. Nevertheless, limitations in the description of the SVT type, history, and long-term management in our registry justify pursuing research on this topic.

## Data Availability

The raw data supporting the conclusions of this article will be made available by the authors, without undue reservation.

## Glossary

### Abbreviations

ACE: angiotensin-converting enzyme
ACEi: angiotensin-converting enzyme inhibitor
AF: atrial fibrillation
ARB: angiotensin receptor blocker
ALAT: alanine aminotransferase
AMI: acute myocardial infarction
ASAT: aspartate aminotransferase
BNP: brain natriuretic peptide
CCTIRS: Comité Consultatif sur le Traitement de l’Information en matière de Recherche dans le domaine de la Santé
CI: confidence interval
CM: cardiomyopathy
CNIL: Commission nationale de l'informatique et des libertés
COPD: chronic obstructive pulmonary disease
CRP: C-reactive protein
CS: cardiogenic shock
DBP: diastolic blood pressure
DOAC: direct oral anticoagulant
ECLS: extracorporeal life support
HF: heart failure
HR: hazard ratio
HTx: heart transplantation
IABP: intra-aortic balloon pump
ICU: intensive care unit
ICCU: intensive cardiac care unit
ICD: implantable cardioverter-defibrillator
IQR: interquartile range MRA, mineralocorticoid receptor antagonist
LVEF: left ventricle ejection fraction
MBP: mean blood pressure
Nt-proBNP: N-terminal-pro hormone BNP
NYHA: New York Heart Association
PSVtdi: peak systolic velocity tissue Doppler imaging
PT: prothrombin time
SBP: systolic blood pressure
SD: standard deviation
STEMI: ST-segment elevation myocardial infarction
SVT: supraventricular tachycardia
TAPSE: tricuspid annular plane systolic excursion
TIC: tachycardia-induced cardiomyopathy
VA: ventricular arrythmias
VAD: ventricular assist device
VIF: variance inflation factor
