# Supplementary material for: One-year outcomes in cardiogenic shock triggered by supraventricular tachycardia: an analysis of the FRENSHOCK multicenter prospective registry
Source: Front Cardiovasc Med. 2023 Sep 5;10:1167738. doi: 10.3389/fcvm.2023.1167738 (PMC10507701; doi:10.3389/fcvm.2023.1167738)
Supplement: Supplementary file 1 [file Datasheet1.docx]

**Supplemental material**

**One-year outcomes in cardiogenic shock triggered by supraventricular tachycardia: an analysis of the FRENSHOCK multicenter prospective registry**, by Miloud Cherbi et al.

**Table S1:** Paired differences of clinical, echocardiographic, and laboratory parameters between initial presentation and after 24h of in-hospital management in the overall population.

**Table S2**: Clinical characteristics at admission in SVT-triggered CS according to coexisting triggers

**Table S3**: Clinical, echocardiographic, and laboratory parameters in SVT-triggered CS according to coexisting triggers

**Table S4:** Paired differences of clinical, echocardiographic, and laboratory parameters between initial presentation and

after 24h of in-hospital management in the SVT group.

**Table S5:** Independent predictors of each outcome in multivariate analysis in the SVT-triggered population (n = 100)**.**

**Table S6:** In-hospital management in SVT-triggered CS according to coexisting triggers.

**Table S7**: Antiarrhythmic therapies in SVT-triggered CS according to coexisting triggers.

**Figure S1**: Secondary outcomes in the SVT-triggered group according to coexisting triggers and previous cardiomyopathy.

| **Table S1 Paired differences of clinical, echocardiographic, and laboratory parameters between initial presentation and  after 24h of in-hospital management in the overall population.** | | | | | | |
| --- | --- | --- | --- | --- | --- | --- |
|  | **SVT group (n = 100)** | | **Non-SVT group (n = 669)** | | |  |
|  | **Median difference  [95% CI]** | **p value** | **Median difference  [95% CI]** | **p value** | |  |
| Clinical presentation  SBP, mmHg  DBP, mmHg  MBP, mmHg | 3.6 [-1.5, 9]  0 [-4.5, 4]  1.5 [-3.5, 5.5] | 0.15  0.93  0.52 | 4 [1.5, 6]  -2 [-3.5, -0.5]  0.5 [-1, 2] | | < 0.01  0.01  0.67 |  |
| Blood tests  Sodium, mmol/L  Potassium, mmol/L  Creatinin, μmol/L  Bilirubin, mg/L  Haemoglobin, g/dL  Arterial blood lactates, mmol/L  PT, %  Nt-proBNP, pg/mL  BNP, pg/mL | 1.5 [0.5, 3]  -0.8 [-1, -0.5]  -5 [-11, 2]  1.5 [-1, 5]  -0.4 [-1, -0.01]  -1.4 [-2, -0.6]  2.5 [-0.5, 5.5]  -2,099.1 [-4,805, 198]  -617 [-2,889.5, 945.5] | < 0.01  < 0.01  0.16  0.27  0.03  < 0.01  0.12  0.07  0.41 | 1 [0.5, 1.5]  -0.6 [-0.7, -0.4]  -2.5 [-5, -0.01]  1.5 [0.5, 2.4]  -1 [-1, -0.75]  -1.5 [-1.8, -1.2]  0 [-1, 1.5]  69.5 [-2,479, 2,279.5]  -125.9 [-368.5, 57.5] | | < 0.01  < 0.01  0.03  < 0.01  < 0.01  < 0.01  0.71  0.94  0.18 |  |
| Echocardiography   LVEF, %  TAPSE, mm  PSVtdi, cm/s | 3.5 [-0.01, 5]  2.5 [1, 4]  1.5 [0.5, 2.5] | 0.06  0.14  0.03 | 3.5 [2.5, 5]  1 [-0.5, 2]  1 [-0.01, 2] | | < 0.01  0.14  0.13 |  |

BNP = Brain natriuretic peptide, DBP = diastolic blood pressure, IQR = interquartile range, LVEF = left ventricular ejection fraction, MBP = mean blood pressure, PSVtdi = peak systolic velocity tissue Doppler imaging, PT = prothrombin time, SBP = systolic blood pressure, SD = standard deviation, TAPSE = tricuspid annular plane systolic excursion

| **Table S2 Clinical characteristics at admission in SVT-triggered CS according to coexisting triggers.** | | | | |  | |
| --- | --- | --- | --- | --- | --- | --- |
|  | **CS with SVT as exclusive trigger (n = 65)** | **CS with SVT as non-exclusive trigger (n = 35)** | **p value** |  | |  |
| Age, mean ± SD, years | 67.2 ± 11.7 | 63.7 ± 13.6 | 0.13 |  | |  |
| Male, n (%) | 53 (81.5) | 38 (80) | 1 |  | |  |
| Body mass index, mean ± SD, kg/m² | 27 ± 5.3 (n = 64) | 25.6 ± 4.8 | 0.23 |  | |  |
| Risk factors, n (%)  Diabetes mellitus  Hypertension  Dyslipidemia  Current smoker | 19 (29.2)  28 (43.1)  27 (41.5)  13 (20) | 8 (22.9)  18 (51.4)  10 (28.6)  13 (37.1) | 0.65  0.35  0.29  0.1 |  | |  |
|  |  |  |  |  | |  |
|  |  |  |  |  | |  |
|  |  |  |  |  | |  |
| Medical history, n (%)  Peripheral artery disease  Myocardial revascularization  Chronic kidney failure  ICD  COPD  Active cancer  Stroke | 7 (10.8)  25 (38.5)  21 (32.3)  18 (27.7)  5 (7.7)  0 (0)  9 (13.8) | 8 (22.9)  7 (20)  11 (31.4)  5 (14.3)  3 (8.6)  5 (14.3)  5 (14.3) | 0.19  0.09  1  0.2  1  < 0.01  1 |  | |  |
|  |  |  |  |  | |  |
|  |  |  |  |  | |  |
|  |  |  |  |  | |  |
|  |  |  |  |  | |  |
|  |  |  |  |  | |  |
|  |  |  |  |  | |  |
| History of cardiac disease, n (%)  All causes  Ischemic  Hypertrophic  Toxic  Dilated  Valvular  Hypertensive | 51 (78.5)  30 (46.2)  2 (3.1)  2 (3.1)  12 (18.5)  11 (16.9)  0 (0) | 26 (74.3)  9 (25.7)  0 (0)  5 (14.3)  3 (8.6)  4 (11.4)  1 (2.9) | 0.82  0.07  0.76  0.09  0.3  0.66  0.75 |  | |  |
|  |  |  |  |  | |  |
|  |  |  |  |  | |  |
|  |  |  |  |  | |  |
|  |  |  |  |  | |  |
| NYHA functional status, n (%)  I  II  III  IV | 7 (10.8)  26 (40)  24 (36.9)  8 (12.3) | 9 (26.5) (n = 34)  10 (29.4) (n = 34)  11 (32.4) (n = 34)  4 (11.8) (n = 34) | 0.24 |  | |  |
|  |  |  |  |  | |  |
|  |  |  |  |  | |  |
|  |  |  |  |  | |  |
| Previous medications, n (%)  Aspirin  P2Y12 inhibitors  Vitamin K antagonist  DOAC  ACE inhibitors  Sacubitril/valsartan  Statins  Beta blockers  Loop diuretics  Aldosterone antagonist  Thiazide diuretics  Non-dihydropyridine CCB  Amiodarone  Other antiarrhythmic | 30 (46.2)  7 (10.8)  21 (32.3)  14 (21.5)  32 (49.2)  2 (3.3) (n = 61)  29 (82.9)  35 (53.8)  43 (66.2)  16 (24.6)  3 (4.6)  2 (3.1)  22 (33.8)  6 (9.5) (n = 63) | 7 (20)  3 (8.6)  11 (31.4)  8 (22.9)  11 (31.4)  2 (5.9) (n = 34)  10 (28.6)  16 (45.7)  22 (62.9)  10 (28.6)  2 (5.7)  1 (2.9)  13 (54.2) (n = 24)  1 (3) (n = 33) | 0.02  1  1  1  0.13  0.94  0.18  0.57  0.91  0.85  1  1  0.83  0.45 |  | |  |
|  |  |  |  |  | |  |
|  |  |  |  |  | |  |
|  |  |  |  |  | |  |
|  |  |  |  |  | |  |
|  |  |  |  |  | |  |
|  |  |  |  |  | |  |
|  |  |  |  |  | |  |
|  |  |  |  |  | |  |
|  |  |  |  |  | |  |
|  |  |  |  |  | |  |
|  |  |  |  |  | |  |
|  |  |  |  |  | |  |
|  |  |  |  |  | |  |

ACE = angiotensin-converting enzyme, CCB = calcium channel blocker, COPD = chronic obstructive pulmonary disease, CS = cardiogenic shock, DOAC = direct oral anticoagulant, ICD = implantable cardioverter-defibrillator, NYHA = New York Heart Association, SD = standard deviation, SVT = supra-ventricular tachycardia

| **Table S3 Clinical, echocardiographic, and laboratory parameters in SVT-triggered CS according to** **coexisting triggers**. | | | | | |
| --- | --- | --- | --- | --- | --- |
|  | **CS with SVT as exclusive trigger (n = 65)** | **CS with SVT as non-exclusive trigger (n = 35)** | **p value** |  |  |
| Clinical presentation at admission  SBP, mean ± SD, mmHg  DBP, mean ± SD, mmHg  MBP, mean ± SD, mmHg  Mottling, n (%)  Cardiac arrest, n (%) | 107 ± 28.3  69.8 ± 18.7 (n = 64)  82.2 ± 21.3 (n = 64)  24 (40.7) (n = 59)  6 (9.2) | 97.7 ± 25.8  58.5 ± 16.6  71.3 ± 18.5 14 (45.2) (n = 31)  1 (2.9) | 0.06  < 0.01  0.01  0.85  0.44 |  |  |
| Blood tests at admission, median (IQR)  Sodium, mmol/L  Potassium, mmol/L  Creatinin, μmol/L  Bilirubin, mg/L  Arterial blood lactates, mmol/L  PT, %  Nt-proBNP, pg/mL  BNP, pg/mL  CRP, mg/L | 136 (133 – 140)  4.45 (4 – 5) (n = 50)  140 (114 – 209)  24.5 (17 – 41.3) (n = 48)  2.9 (2 – 3.6) (n = 60)  44 (28.3 – 60.3) (n = 64)  8,940 (5,825.5 – 13,150) (n = 15)  1,716 (795 – 2,590) (n = 21)  23 (11.3 – 48) (n = 30) | 133 (130 – 137)  4.33 (4 – 5) (n = 32)  154 (101.5 – 217.5)  24.5 (15 – 39.5) (n = 30)  3 (2 – 4.8) (n = 34)  46 (32.5 – 67)  18,100.5 (12,232.8 – 29,744.5) (n = 14)  849 (383.5 – 2,613.5) (n = 10)  31.5 (13.5 – 64.8) (n = 24) | 0.01  0.9  0.93  0.59  0.41  0.32  0.13  0.37  0.42 |  |  |
| Baseline echocardiography  LVEF, mean ± SD, %  TAPSE, median (IQR), mm  Severe mitral regurgitation, n (%)  Severe aortic stenosis, n (%)  Severe aortic regurgitation, n (%) | 22.7 ± 11.3 (n = 64)  10 (9.5 – 13.5) (n = 27)  16 (25.4) (n = 63)  4 (6.3) (n = 64)  0 (0) (n = 64) | 25.9 ± 13.3 (n = 34)  11 (10.3 – 11.8) (n = 10)  6 (17.6) (n = 34)  1 (2.9) (n = 34)  1 (2.9) (n = 34) | 0.19  0.74  0.54  0.82  0.75 |  |  |

ALAT = alanine aminotransferase, ASAT = aspartate aminotransferase, BNP = Brain natriuretic peptide, CRP = C-reactive protein, CS = cardiogenic shock, DBP = diastolic blood pressure, IQR = interquartile range, LVEF = left ventricular ejection fraction, MBP = mean blood pressure, Nt-proBNP = N-terminal-pro hormone BNP, PSVtdi = peak systolic velocity tissue Doppler imaging, PT = prothrombin time, SBP = systolic blood pressure, SD = standard deviation, SVT = supra-ventricular tachycardia, TAPSE = tricuspid annular plane systolic excursion

| **Table S4 Paired differences of clinical, echocardiographic, and laboratory parameters between initial presentation and  after 24h of in-hospital management in the SVT group.** | | | | | | |
| --- | --- | --- | --- | --- | --- | --- |
|  | **CS with SVT as exclusive trigger  (n = 65)** | | **CS with SVT as non-exclusive trigger  (n = 35)** | | |  |
|  | **Median difference  [95% CI]** | **p value** | **Median difference  [95% CI]** | **p value** | |  |
| Clinical presentation  SBP, mmHg  DBP, mmHg  MBP, mmHg | 2 [-4.5, 8]  -1.5 [-7.5, 4]  -0.5 [-7, 5] | 0.47  0.59  0.86 | 7.5 [-3, 17]  2.5 [-3.5, 9]  5 [-2, 12] | | 0.16  0.39  0.14 |  |
| Blood tests  Sodium, mmol/L  Potassium, mmol/L  Creatinin, μmol/L  Bilirubin, mg/L  Haemoglobin, g/dL  Arterial blood lactates, mmol/L  PT, %  Nt-proBNP, pg/mL  BNP, pg/mL | 1 [-0.5, 2.5]  -0.9 [-1.25, -0.4]  -3.5 [-11, 7]  1.5 [-1.5, 5]  -0.4 [-1, 0.05]  -1 [-2, 0.1]  2.5 [-1.5, 6]  503.9 [-2,902, 4000]  -987 [-3,653, 88] | 0.14  < 0.01  0.41  0.36  0.03  0.12  0.23  0.86  0.28 | 2.5 [1, 4.5]  -0.7 [-1.07, -0.15]  -7 [-17.5, 2]  2 [-5.5, 14]  -0.5 [-1.1, -0.01]  -1.7 [-3.3, -0.5]  2.5 [-3.5, 8]  -3,643.2 [-8,270.5, -773.5]  -35.5 [-1,355.5, 1,948.5] | | < 0.01  < 0.01  0.12  0.6  0.09  0.02  0.39  0.03  1 |  |
| Echocardiography   LVEF, %  TAPSE, mm  PSVtdi, cm/s | 5 [2.5, 8]  1 [-1, 3]  NA | < 0.01  0.59 | 0 [-12.5, 7.5]  NA  NA | | 0.92 |  |

BNP = Brain natriuretic peptide, DBP = diastolic blood pressure, IQR = interquartile range, LVEF = left ventricular ejection fraction, MBP = mean blood pressure, PSVtdi = peak systolic velocity tissue Doppler imaging, PT = prothrombin time, SBP = systolic blood pressure, SD = standard deviation, TAPSE = tricuspid annular plane systolic excursion

| **Table S5** **Independent predictors of each outcome in multivariate analysis in the SVT-triggered population (n = 100)** | | | | |
| --- | --- | --- | --- | --- |
|  | **Characteristics** | **OR (95% CI)** | **p value** |  |
| 1-month mortality    1-year mortality | Age  Male sex  Current smoker  ICD  Age  Chronic kidney failure Active cancer | 1.01 (1.004 – 1.02)  0.74 (0.59 – 0.94)  1.34 (1.03 – 1.73)  1.3 (1.04 – 1.63)  1.007 (1.004 – 1.01)  1.14 (1.04 – 1.26)  1.31 (1.12 – 1.53) | < 0.01  0.01  0.03  0.02  < 0.01  < 0.01  < 0.01 |  |
| Rehospitalizations at 1 year | Age | 0.987 (0.979 – 0.996) | < 0.01 |  |
| Heart transplantation or ventricular assistance at 1 year | Age  History of ischemic heart disease  History of dilated heart disease | 0.992 (0.987 – 0.998)  1.22 (1.06 – 1.39)  1.23 (1.03 – 1.47) | < 0.01  < 0.01  0.02 |  |
| Mortality or heart transplantation or ventricular assistance at 1 year | Age | 1.001 (1.0001 – 1.02) | 0.048 |  |

CI = confidence interval, ICD = implantable cardioverter-defibrillator, OR = odds ratio, SVT = supraventricular tachycardia

| **Table S6 In-hospital management in SVT-triggered CS according to** **coexisting triggers.** | | | | |
| --- | --- | --- | --- | --- |
|  | **CS with SVT as exclusive trigger (n = 65)** | **CS with SVT as non-exclusive trigger (n = 35)** | **p value** |  |
| Medications used, n (%)  Dobutamine or norepinephrine or levosimendan  Dobutamine  Norepinephrine  Levosimendan | 53 (81.5)  47 (72.3)  26 (40)  9 (13.8) | 34 (97.1)  32 (91.4)  16 (45.7)  4 (11.4) | 0.06  0.048  0.73  0.98 |  |
| Respiratory support, n (%)  Non-invasive  Invasive | 17 (26.2)  22 (33.8) | 6 (17.1)  9 (25.7) | 0.44  0.54 |  |
| Short-term mechanical circulatory support, n (%)  IABP  Impella  ECLS | 2 (3.1)  2 (3.1)  11 (16.9) | 1 (2.9)  1 (2.9)  1 (2.9) | 1  1  0.08 |  |
| Renal replacement therapy, n (%) | 12 (18.5) | 2 (5.7) | 0.15 |  |

CS = cardiogenic shock, ECLS = extracorporeal life support, IABP = intra-aortic balloon pump, SVT = supraventricular tachycardia

| **Table S7 Antiarrhythmic therapies in SVT-triggered CS according to** **coexisting triggers.** | | | | |
| --- | --- | --- | --- | --- |
|  | **CS with SVT as exclusive trigger (n = 65)** | **CS with SVT as non-exclusive trigger (n = 35)** | **p value** |  |
| Betablockers, n (%)  Initial care  24h  Discharge  1 year | 35 (53.8)  11 (18) (n = 61)  26 (54.2) (n = 48)  19 (57.6) (n = 33) | 16 (45.7)  2 (6.3) (n = 32)  15 (65.2) (n = 23)  9 (64.3) (n = 14) | 0.57  0.21  0.53  0.92 |  |
| Non-dihydropyridine calcium channel blockers, n (%)  Initial care  24h  Discharge  1 year | 2 (3.1)  1 (1.6) (n = 61)  0 (0) (n = 45)  2 (6.9) (n = 29) | 1 (2.9)  0 (0) (n = 31)  0 (0) (n = 23)  0 (0) (n = 12) | 1  1  N/A  0.89 |  |
| Amiodarone, n (%)  Initial care  24h  Discharge  1 year | 22 (33.8)  36 (60) (n = 60)  23 (51.1) (n = 45)  10 (33.3) (n = 30) | 13 (38.2) (n = 34)  18 (56.3) (n = 32)  9 (39.1) (n = 23)  2 (16.7) (n = 12) | 0.83  0.9  0.5  0.48 |  |
| Other anti-arrhythmic, n (%)  Initial care  24h  Discharge  1 year | 6 (9.5)  4 (6.9) (n = 58)  3 (6.5) (n = 46)  5 (17.9) (n = 28) | 1 (3) (n = 33)  5 (15.6) (n = 32)  2 (8.7) (n = 23)  1 (8.3) (n = 12) | 0.45  0.34  1  0.77 |  |
| ICD implantation, n (%) | 3 (5) (n = 60) | 1 (3.2) (n = 31) | 1 |  |
| SVT catheter ablation, n (%) | 8 (13.3) (n = 60) | 2 (6.5) (n = 31) | 0.52 |  |

ICD = Implantable cardioverter-defibrillator, SVT = supra-ventricular tachycardia


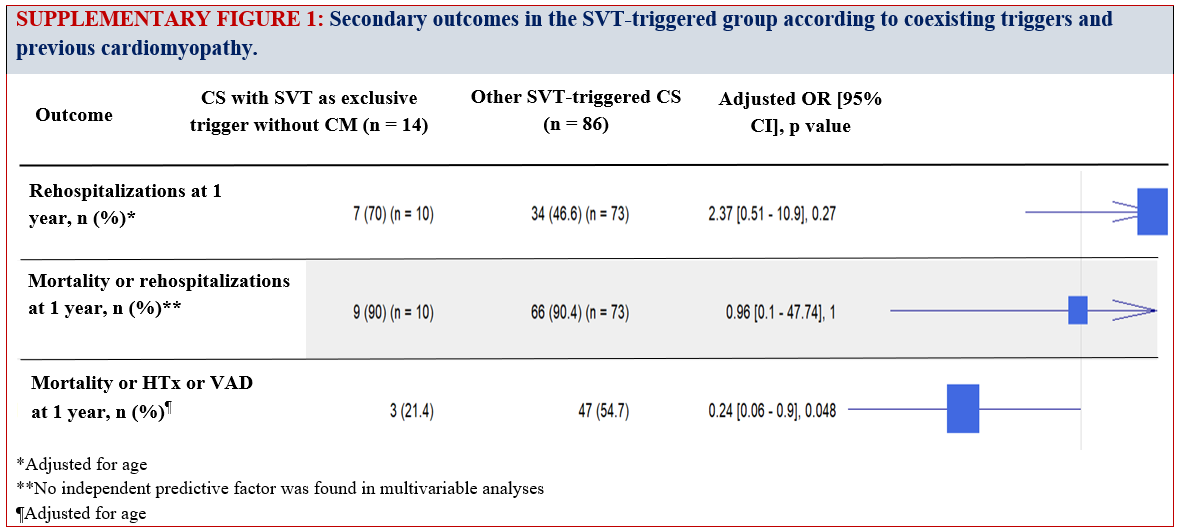


**Figure S1: Secondary outcomes in the SVT-triggered group according to coexisting triggers and previous cardiomyopathy.**

HTx and VAD comparison was not presented since there were no events in the TIC group, with incalculable OR.

Each adjusted outcome analysis included significant characteristics found as independent predictive factors in multivariable analyses and used as fixed covariates.

CM = cardiomyopathy, CS = cardiogenic shock, HTx = heart transplantation, SVT = supraventricular tachycardia, VAD = ventricular assistance device
